# Eye Manifestations of Shprintzen–Goldberg Craniosynostosis Syndrome: A Case Report and Systematic Review

**DOI:** 10.1155/2020/7353452

**Published:** 2020-08-19

**Authors:** Jamie H. Choi, Rachel Li, Rachel Gannaway, Tahnee N. Causey, Anna Harrison, Natario L. Couser

**Affiliations:** ^1^Virginia Commonwealth University School of Medicine, Richmond, VA, USA; ^2^Department of Human and Molecular Genetics, Division of Clinical Genetics, Virginia Commonwealth University School of Medicine, Richmond, VA, USA; ^3^Department of Ophthalmology, Virginia Commonwealth University School of Medicine, Richmond, VA, USA; ^4^Department of Pediatrics, Virginia Commonwealth University School of Medicine, Richmond, VA, USA

## Abstract

Shprintzen–Goldberg craniosynostosis syndrome (SGS) is a rare autosomal dominant condition that was first documented in literature in 1982. The disorder is caused by pathogenic variants in the proto-oncogene *SKI* gene, a known suppressor of TGF-*β* activity, located on chromosome 1p36. There is considerable phenotypic overlap with Marfan and Loeys–Dietz syndromes. Common clinical features of SGS include craniosynostosis, marfanoid habitus, hypotonia, dysmorphic facies, cardiovascular anomalies, and other skeletal and connective tissue abnormalities. Ocular manifestations may include hypertelorism, downslanting palpebral fissures, proptosis, myopia, and ectopia lentis. We describe a 25-year-old male with the syndrome. Genetic analysis revealed a novel c.350G>A (p.Arg117His) *de novo* variant, which was predicted to be pathogenic by the CTGT laboratory. The patient presented with dysmorphic features, marfanoid habitus, severe joint contractures, mitral valve insufficiency, aortic root dilatation, and a history of seizures. His ocular manifestations included hypertelorism, downslanting palpebral fissures, bilateral ptosis, and high myopia. Ophthalmic manifestations are an integral component of the syndrome; however, they have not been well characterized in the literature. From a systematic review of previously published cases to date, we summarize the eye and ocular adnexa manifestations reported.

## 1. Introduction

Shprintzen–Goldberg craniosynostosis syndrome (SGS; MIM #182212) is an autosomal dominant disorder comprising craniosynostosis and marfanoid habitus, along with other skeletal, neurological, cardiovascular, and connective tissue anomalies. Though first described by Sugarman and Vogel in 1981, it was not documented until 1982 by Shprintzen and Goldberg in two unrelated patients [1]. Since its initial description, less than 50 cases of SGS have been reported [2].

There is phenotypic overlap between SGS and Marfan and Loeys–Dietz syndromes, which all share similar skeletal and cardiovascular anomalies. Increased TGF-*β* signaling has been implicated in the pathogenesis of the latter two conditions [3]. Pathogenic variants in the proto-oncogene *SKI*, a known repressor of TGF-*β* activity, are currently the only known cause of SGS [2, 3]. All reported pathogenic variants in *SKI*-positive Shprintzen–Goldberg cases were found to reside within exon 1, from nucleotide c.63 to c.351 (p.21–p.117) [4].

The majority of cases are due to *de novo* variant persons with no family history of SGS or related conditions. There have been rare reports of SGS where the altered gene was inherited from an unaffected parent who possessed the mutation only in their germline cells. Other genes may be implicated in SGS, such as that of fibrillin-1 (*FBN1*) in Marfan syndrome and *TGFBR* in Loeys–Dietz syndrome, but further research is warranted to better understand their involvement in this condition [2].

In addition to craniosynostosis, people with SGS are born with several key dysmorphic craniofacial features, such as dolichocephaly, low-set ears, micrognathia, a high-arched palate, craniosynostosis, maxillary hypoplasia, and postrotated ears [4, 5]. Common ocular findings include hypertelorism, downslanting palpebral fissures, myopia, proptosis, and strabismus [6]. As infants, patients present with delayed developmental and motor milestones, hypotonia, and varying degrees of developmental delay, all of which distinguish SGS from Marfan syndrome [5]. While patients with Loeys–Dietz syndrome have normal intelligence, up to 15% can show cognitive delays, especially if related to a chromosomal deletion. Skeletal findings in SGS include dolichostenomelia, arachnodactyly, pectus chest deformities, joint contractures, joint hypermobility, vertebral abnormalities, and marfanoid habitus [4, 6]. Cardiovascular anomalies include mitral valve prolapse and aortic root dilatation, which is more pronounced in Loeys–Dietz syndrome. Several brain abnormalities have been described, including hydrocephalus, ventricular dilation, and Chiari I malformation [4]. In this report, we summarize the eye and ocular adnexa manifestations reported to date of SGS, and additionally present an adult male with the syndrome.

## 2. Case Report

The patient, now a 25-year-old African American male, was born full-term at 41 weeks via spontaneous vaginal delivery to a G3P3 mother, weighing 3.71 kg. There were no pregnancy or delivery-related complications.

**Figure 1:**
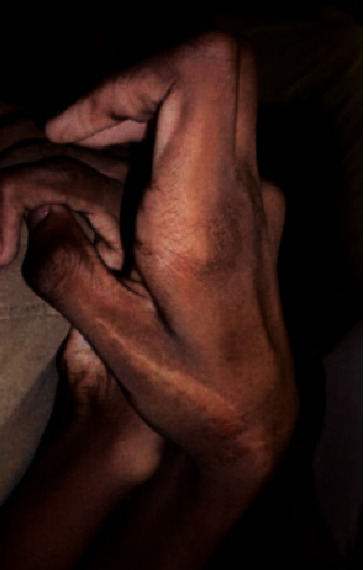
Joint contractures present in bilateral hands.

The patient was first assessed by our care team at the age 16. Height and weight were both above the 95^th^ percentile. Head circumference was at the 50^th^ percentile. On exam, the patient appeared tall but physically disabled due to multiple joint contractures. Dysmorphic features included dolichocephaly, shallow orbits with mild ptosis (margin reflex distance, MRD1, was 2 mm in both eyes), hypertelorism (outer canthal distance of 10 cm and inner canthal distance of 4 cm), downslanting palpebral fissures, broad-based nasal bridge, midface hypoplasia with prognathism, high-arched palate, low-set ears, and pectus carinatum. Arachnodactyly was present with ulnar camptodactyly and severe contractures of all digits (Figure 1). A decreased range of motion of the major joints was present. A comprehensive two-dimensional Doppler color flow echocardiogram was notable for normal intracardiac anatomy and left ventricular function, but a mildly dilated aortic annulus at 3 cm.

At age 23, the patient underwent CT angiograms of his abdomen, pelvis, chest, and neck. Notable findings included mild ectasia of the left common iliac artery, aneurysmal dilation of the aortic root at 4.3 cm, cardiomegaly, bilateral apical bullous disease, dilatation of the proximal pulmonary trunk above the pulmonic valve up to 4.5 cm, and 4 mm left subluxation of C1 and C2. There were no other aneurysms or arterial tortuosity found on imaging.

**Figure 2:**
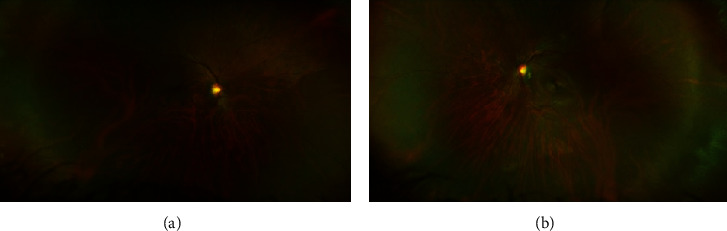
The discharge curves for release structures of the PBG hydropower station.

At his last examination at age 25, eye and ocular adnexal abnormalities included high myopia in both eyes, hypertelorism, downslanting palpebral fissures, bilateral ptosis, and 4 prism diopters of exophoria. His refraction measured −14.00 + 3.25 × 130 and −14.75 + 2.25 × 065 in the right and left eyes, respectively. With correction, his visual acuity measured 20/25 in each eye. There was no apparent lens dislocation in either eye. Fundus exam was notable for tilted optic nerves, and a scleral crescent was more prominent temporally in both eyes (Figure 2). There were no obvious cornea or lenticular changes on slit-lamp examination; as such, we suspect that his myopia is likely related to dysregulated globe elongation. Axial length was not recorded and, as a result, cannot be definitively stated. His additional medical conditions included dilated cardiomyopathy, seizure disorder, bullous lung disease, gastroesophageal reflux disease, and scoliosis. Repeat echocardiogram was notable for a mildly decreased left ventricular systolic function, with an ejection fraction of 46.6%. Family history was notable for myopia reportedly present in his mother.

Genetic testing included a normal karyotype. The patient tested negative for Angelman syndrome, Williams syndrome, and Fragile X. Testing for Marfan and other thoracic aortic aneurysm syndromes were then pursued. He was found to have a benign FBN1 variant of uncertain significant at 15q13.1-q13.2, inherited from an unaffected parent. As the remaining tests returned negative, the patient and his family agreed to pursue sequencing of the SKI gene for SGS. The patient had normal sequencing results for *FBN2*, *MYH11*, *ACTA2*, *TGFBR1*, and *TGFBR2* mutation analysis. Sequencing of the *SKI* gene later revealed a c.3502G>A (p.Arg117His) novel transition in codon 1, which was predicted to be pathogenic.

## 3. Methods

We performed a systematic review of the literature to summarize ocular abnormalities in individuals with Shprintzen–Goldberg syndrome. A PubMed/Medline search of “Shprintzen–Goldberg” OR “*SKI* gene” returned a total of 109 articles. No articles were excluded based on the year published or language. Within the articles, we identified cases of Shprintzen–Goldberg syndrome due to the *SKI* gene. References were reviewed to identify other articles that did not appear in our original search.

## 4. Discussion

From our systematic review, we found 6 articles describing 44 reported cases of Shprintzen–Goldberg craniosynostosis syndrome due to pathologic variants in the *SKI* gene on chromosome 1p36. Including our patient, ninety-seven percent (*n* = 44) of patients reported with this syndrome had ophthalmologic abnormalities (Table 1). The most common ocular findings were hypertelorism (*n* = 43), downslanting palpebral fissures (*n* = 37), proptosis (*n* = 33), and myopia (*n* = 10).

Forty-four total variants have been recorded in literature. Of those, there are twenty-one unique pathogenic variants reported within exon 1 of the *SKI* gene. The most common pathogenic variant is the c.94C>G (p.Leu32Val) variant (*n* = 6). There were 5 reported cases of a deletion located within c.280_291del (p.Ser94_Ser97del), which consisted of members from the same family; all inherited the deletion in an autosomal dominant fashion and notably had an absence of craniosynostosis, a typical feature of SGS.

The majority of cases were inherited *de novo* (*n* = 26). Carmignac et al. [7] reported 5 cases within the same family, as previously mentioned (Supplemental 1(b): family 3). Another family consists of 3 affected siblings, but the parents were asymptomatic due to germline mosaicism (Supplemental 1(b): family 4). Variable expressivity can exist in SGS, even amongst members of the same family.

Au et al. [4] described a 46-year-old woman with SGS and prominent ocular manifestations. It is the only case reporting findings such as retinal detachment and proliferative vitreoretinopathy; the patient also had hypertelorism, myopia, downslanting palpebral fissures, proptosis, amblyopia, exotropia, cataracts, and low vision. The pathogenic variant noted, c.103C>T (p.Pro35Ser), is shared by four other patients reported in literature, who were aged 4, 6, 10, and 21. They all were characterized to have hypertelorism, downslanting palpebral fissures, and proptosis. The oldest patient with SGS recorded from our review was a 50-year-old male, who presented with hypertelorism, downslanting palpebral fissures, and proptosis [4].

Mutations to TGF-*β* signaling are implicated in both Marfan and Loeys–Dietz syndromes. Given the clinical overlap between SGS and these particular thoracic aortic aneurysm phenotypes, it is unsurprising that the *SKI* pathogenic variants also affect the TGF-*β* signaling cascade. *Xenopus* embryos, zebrafish, and mice with *in vivo SKI* variants also exhibited changes in the development of the muscle, craniofacial structures, and central or peripheral nervous system resembling those seen in patients with SGS [8]. Though ectopia lentis was reported in three patients, our patient did not exhibit any degree of apparent lens dislocation. Additionally, the *SKI* gene promotes neurogenesis, which may otherwise lead to intellectual disability [8].

SGS variants are clustered within two domains of the SKI gene. The R-SMAD binding domain is involved in TGF-*β* signaling. On the other hand, the Dachshund homology domain (DHD) mediates binding with SNW1 and N-Cor proteins. These proteins are responsible for the transforming activity of the SKI protein in addition to recruitment of transcriptional corepressors. SKI mutations are most likely destroying the binding capacities of these domains [8].

In summary, this case report and review contributes to our current understanding of the relationship between pathologic variants in the *SKI* gene and eye manifestations and provides incentive to further investigate the phenotype.

## Figures and Tables

**Table 1:** Summary of 45 SGS patients with SKI gene mutations. A more detailed description can be found in Supplementary Materials.

Patient	1	2	3	4	5	6	7	8	9	10	11	12	13	14	15	16	17	18	19	20	21	22	23	24	25	26	27	28	29	30	31	32	33	34	35	36	37	38	39	40	41	42	43	44	45
Inh.	dn	dn	dn	dn	dn	dn	dn	dn	dn	dn	dn	dn	dn	dn	dn	dn	dn	dn	dn	dn	dn	nk	dn	dn	dn	nk	nk	nk	ad	dn	dn	nk	nk	nk	dn	dn	dn	dn	nk	dn	gm	gm	dn	dn	dn
Age	43	6	16	12	22	21	2	6	5	4	21	20	42	11	44	13	14	22	22	20	18	16	5	21	10	11	32	20	26	46	3	50	44	5	4	10	10	12	16	9	13	22	nk	10	25
Eye	+	+	+	+	+	+	+	+	+	+	+	+	+	+	+	+	+	+	+	+	+	+	+	+	+	+	+	+	+	+	+	+	+	+	+	+	+	+	+	+	+	+	+	nk	+
Dysm	+	+	+	+	+	+	+	+	+	+	+	+	+	+	+	+	+	+	+	+	+	+	+	+	+	+	+	+	+	+	+	+	+	+	+	+	+	+	+	+	+	+	+	−−	+
Cardiac	+	+	+	+	+	+	+	−	+	+	−	−	−	−	−	−	−	−	−	−	+	−	−	+	+	+	+	−	−	+	−	−	nk	nk	−	−	−	−	+	+	+	−	−	nk	+
MSK	+	+	+	+	+	+	+	+	+	+	+	+	+	+	+	+	+	+	+	+	+	+	+	+	+	+	+	+	+	+	+	+	+	+	+	+	+	+	+	+	+	+	+	+	+
Neuro	+	+	+	+	+	+	+	+	+	+	+	+	+	+	+	+	+	+	+	+	+	+	+	+	+	+	+	+	+	+	+	+	+	+	+	nk	nk	+	nk	nk	nk	nk	+	+	+
DD	+	+	+	+	+	+	+	+	+	+	+	+	+	+	+	+	+	+	+	+	+	+	+	+	+	+	+	+	+	+	+	+	+	+	+	+	+	+	+	+	+	−	+	nk	nk

Inh., inheritance; Eye, ocular findings; Dysm, dysmorphic features; Cardiac, cardiac anomalies; MSK, musculoskeletal anomalies; Neuro, neurological anomalies; DD, developmental delay; ad, autosomal dominant; dn, de novo; gm, germline mosaicism; nk, not known.

## Data Availability

The data used to support this study are publicly available on PubMed.

## Supplementary Materials

Supplementary Materials Supplemental 1. Summary of 45 SGS patients with SKI gene mutations. (a) [3]. (b) [7]. (c) [7]. (d) [4, 8]. (e) [8, 9, 10].
