# Corrigendum to “Eye Manifestations of Shprintzen–Goldberg Craniosynostosis Syndrome: A Case Report and Systematic Review”

**DOI:** 10.1155/2020/4708976

**Published:** 2020-11-11

**Authors:** Jamie H. Choi, Rachel Li, Rachel Gannaway, Tahnee N. Causey, Anna Harrison, Natario L. Couser

**Affiliations:** ^1^Virginia Commonwealth University School of Medicine, Richmond, VA, USA; ^2^Department of Human and Molecular Genetics, Division of Clinical Genetics, Virginia Commonwealth University School of Medicine, Richmond, VA, USA; ^3^Department of Ophthalmology, Virginia Commonwealth University School of Medicine, Richmond, VA, USA; ^4^Department of Pediatrics, Virginia Commonwealth University School of Medicine, Richmond, VA, USA

In the article titled “Eye Manifestations of Shprintzen–Goldberg Craniosynostosis Syndrome: A Case Report and Systematic Review” [1], Figure 2 has been labelled incorrectly. The correct figure legend should read as follows:

Figure 2: Color images (Optos) showing a tilted optic nerve and scleral crescent more prominent temporally in the right (a) and left (b) fundus.
