# Corrigendum #2 to “Eye Manifestations of Shprintzen–Goldberg Craniosynostosis Syndrome: A Case Report and Systematic Review”

**DOI:** 10.1155/2021/9897523

**Published:** 2021-08-28

**Authors:** Jamie H. Choi, Rachel Li, Rachel Gannaway, Tahnee N. Causey, Anna Harrison, Natario L. Couser

**Affiliations:** ^1^Virginia Commonwealth University School of Medicine, Richmond, VA, USA; ^2^Department of Human and Molecular Genetics, Division of Clinical Genetics, Virginia Commonwealth University School of Medicine, Richmond, VA, USA; ^3^Department of Ophthalmology, Virginia Commonwealth University School of Medicine, Richmond, VA, USA; ^4^Department of Pediatrics, Virginia Commonwealth University School of Medicine, Richmond, VA, USA

In the article titled “Eye Manifestations of Shprintzen–Goldberg Craniosynostosis Syndrome: A Case Report and Systematic Review” [1], the authors have identified additional errors that require correction. These errors are resulting from the misinterpretation of data obtained from Doyle et al. [2].

Supplemental Table 1(a) should be corrected as follows: to remove “ectopia lentis” from patients 4, 7, and 8.

In the Discussion section, the sentence, “though ectopia lentis was reported in three patients, our patient did not exhibit any degree of apparent lens dislocation,” should be deleted. In the abstract, “Ocular manifestations may include hypertelorism, downslanting palpebral fissures, proptosis, myopia, and ectopia lentis” should be changed to “Ocular manifestations may include hypertelorism, downslanting palpebral fissures, proptosis, and myopia.”

## Figures and Tables

**Supplemental Table 1:** Summary of 45 SGS patients with SKI gene mutations.

(a)										
Doyle,et al. [2012]										
Patient	1	2	3	4	5	6	7	8	9	10
SKI gene pathogenic variant	c.347G>A(p.Gly116Glu)	c.349G>C(p.Gly117Arg)	c.101G>A(p.Gly34Asp)	c.94C>G(p.Leu32Val)	c.94C>G(p.Leu32Val)	c.100G>A(p.Gly34Ser)	c.100G>T(p.Gly34Cys)	c.103C>T(p.Pro35Ser)	c.283_291del(p.Asp95_Ser 97del)	c.62T>G(p.Leu21Arg)
Inheritance	de novo	de novo	de novo	de novo	de novo	de novo	de novo	de novo	de novo	de novo
Gender	F	M	M	M	F	M	F	M	M	F
Age(Years)	43	6	16	12	22	21	2	6	5	4
Ocular findings	Hypertelorism,down-slanting eyes, proptosis	Hypertelorism,down-slanting eyes	Hypertelorism,down-slanting eyes, proptosis	Hypertelorism,down-slanting eyes, proptosis	Hypertelorism,down-slanting eyes, proptosis	Hypertelorism, proptosis	Hypertelorism,down-slanting eyes, proptosis	Hypertelorism,down-slanting eyes, proptosis	Hypertelorism,down-slanting eyes, proptosis	Hypertelorism,down-slanting eyes, proptosis
Dysmorphic features	+	+	+	+	+	+	+	+	+	+
Cardiac anomalies	Mitral value prolapse, aortic root dilatation	Mitral value prolapse, aortic root dilatation	Mitral value prolapse, aortic root dilatation	aortic root dilatation	aortic root dilatation	aortic root dilatation	aortic root dilatation	-	Mitral value prolapse	Mitral value prolapse, aortic root dilatation, arterial tortuosity
Musculoskeletal anomalies	+	+	+	+	+	+	+	+	+	+
Neurological anomalies	+	+	+	+	+	+	+	+	+	+
Developmental delay	+	+	+	+	+	+	+	+	+	+
Other	Splenic artery aneurysm	Broad/bifiduvula	Club foot deformity	Cleft palate			Cleft palate	Cleft palate, Club foot deformity, Splenic artery aneurysm with spontaneous rupture		Broad/bifiduvula
