# Supplementary material for: Eye Manifestations of Shprintzen–Goldberg Craniosynostosis Syndrome: A Case Report and Systematic Review
Source: Case Rep Genet. 2020 Aug 19;2020:7353452. doi: 10.1155/2020/7353452 (PMC7895601; doi:10.1155/2020/7353452)
Supplement: Supplementary Materials — Supplemental 1. Summary of 45 SGS patients with SKI gene mutations. (a) [3]. (b) [7]. (c) [7]. (d) [4, 8]. (e) [8, 9, 10]. [file 7353452.f1.docx]

Supplemental 1: Summary of 45 SGS patients with SKI gene mutations

(a)

|  | Doyle, et al. [2012] | | | | | | | | | |
| --- | --- | --- | --- | --- | --- | --- | --- | --- | --- | --- |
| Patient | 1 | 2 | 3 | 4 | 5 | 6 | 7 | 8 | 9 | 10 |
| SKI gene pathogenic  variant | c.347G>A  (p.Gly116Glu) | c.349G>C  (p.Gly117Arg) | c.101G>A  (p.Gly34Asp) | c.94C>G  (p.Leu32Val) | c.94C>G  (p.Leu32Val) | c.100G>A  (p.Gly34Ser) | c.100G>T  (p.Gly34Cys) | c.103C>T  (p.Pro35Ser) | c.283_291del  (p.Asp95_Ser97del) | c. 62T>G  (p.Leu21Arg) |
| Inheritance | de novo | de novo | de novo | de novo | de novo | de novo | de novo | de novo | de novo | de novo |
| Gender | F | M | M | M | F | M | F | M | M | F |
| Age (years) | 43 | 6 | 16 | 12 | 22 | 21 | 2 | 6 | 5 | 4 |
| Ocular findings | hypertelorism, down-slanting eyes, proptosis | hypertelorism, down-slanting eyes | hypertelorism, down-slanting eyes, proptosis | hypertelorism, down-slanting eyes, proptosis, ectopia lentis | hypertelorism, down-slanting eyes, proptosis | hypertelorism, proptosis | hypertelorism, down-slanting eyes, proptosis, ectopia lentis | hypertelorism, down-slanting eyes, proptosis, ectopia lentis | hypertelorism, down-slanting eyes, proptosis | hypertelorism, down-slanting eyes, proptosis |
| Dysmorphic features | + | + | + | + | + | + | + | + | + | + |
| Cardiac anomalies | mitral valve prolapse, aortic root dilatation | mitral valve prolapse, aortic root dilatation | mitral valve prolapse, aortic root dilatation | aortic root dilatation | aortic root dilatation | aortic root dilatation | aortic root dilatation | - | mitral valve prolapse | mitral valve prolapse, aortic root dilatation, arterial tortuosity |
| Musculoskeletal anomalies | + | + | + | + | + | + | + | + | + | + |
| Neurological anomalies | + | + | + | + | + | + | + | + | + | + |
| Developmental delay | + | + | + | + | + | + | + | + | + | + |
| Other | splenic artery aneurysm | broad/bifid uvula | club foot deformity | cleft palate |  |  | cleft palate | cleft palate, club foot deformity, splenic artery aneurysm with spontaneous rupture |  | broad/bifid uvula |

(b)

|  | Carmignac, et al. [2012] | | | | | | | | | |
| --- | --- | --- | --- | --- | --- | --- | --- | --- | --- | --- |
|  | Family 1 | Family 2 | Family 3 | | | | | Family 4 | | |
| Patient | 11 | 12 | 13 | 14 | 15 | 16 | 17 | 18 | 19 | 20 |
| SKI gene pathogenic  variant | c.100G>T  (p.Gly34Cys) | c.94C>G  (p.Leu32Val) | c.280_291del  (p.Ser94_Ser97del) | c.280_291del  (p.Ser94_Ser97del) | c.280_291del  (p.Ser94_Ser97del) | c.280_291del  (p.Ser94_Ser97del) | c.280_291del  (p.Ser94_Ser97del) | c.101G>T  (p.Gly34Val) | c.101G>T  (p.Gly34Val) | c.101G>T  (p.Gly34Val) |
| Inheritance | de novo | de novo | AD | AD | AD | AD | AD | AD, GM | AD, GM | AD, GM |
| Gender | F | F | M | F | F | F | M | F | F | F |
| Age (years) | 21 | 20 | 42 | 11 | 44 | 13 | 14 | 22 | 22 | 20 |
| Ocular findings | hypertelorism, proptosis, down-slanting eyes | hypertelorism, proptosis, down-slanting eyes | hypertelorism, proptosis, down-slanting eyes, myopia | hypertelorism, down-slanting eyes, myopia | hypertelorism, proptosis, down-slanting eyes, myopia | hypertelorism, proptosis, down-slanting eyes, myopia | hypertelorism, down-slanting eyes | hypertelorism, proptosis, down-slanting eyes, myopia | hypertelorism, proptosis, down-slanting eyes, myopia | hypertelorism, down-slanting eyes |
| Dysmorphic features | + | + | + | + | + | + | + | + | + | + |
| Cardiac anomalies | - | - | - | - | - | - | - | - | - | - |
| Musculoskeletal anomalies | + | + | + | + | + | + | + | + | + | + |
| Neurological anomalies | + | + | craniosynostosis  absent | craniosynostosis  absent | craniosynostosis  absent | craniosynostosis  absent | craniosynostosis  absent | + | + | + |
| Developmental delay | + | + | + | + | + | + | + | + | + | + |
| Other | loss of subcutaneous fat |  |  |  |  |  |  | hernias,  loss of  subcutaneous fat | hernias,  loss of subcutaneous fat | hernias |

Abbreviations used: AD, autosomal dominant; GM, germline mosaicism

(c)

|  | Carmignac, et al. [2012] | | | | | | | | |  |
| --- | --- | --- | --- | --- | --- | --- | --- | --- | --- | --- |
|  | Family 5 | Family 6 | Family 7 | Family 8 | Family 9 | Family 10 | Family 11 | Family 12 | Family 13 |  |
| Patient | 21 | 22 | 23 | 24 | 25 | 26 | 27 | 28 | 29 |  |
| SKI gene pathogenic  variant | c.104C>A  (p.Pro35Gln) | c.94C>G  (p.Leu32Val) | c.283_291del  (p.Asp95_Ser  97del) | c.103C>T  (p.Pro35Ser) | c.95T>C  (p.Leu32Pro) | c.100G>A  (p.Gly34Ser) | c.94C>G  (p.Leu32Val) | c.92C>T  (p.Ser31Leu) | - |  |
| Inheritance | de novo |  | de novo | de novo | de novo |  |  |  | AD |  |
| Gender | M | M | F | F | F | F | M | M | M |  |
| Age (years) | 18 | 16 | 5 | 21 | 10 | 11 | 32 | 20 | 26 |  |
| Ocular findings | hypertelorism, proptosis, down-slanting eyes | hypertelorism, down-slanting eyes | hypertelorism, proptosis, down-slanting eyes | hypertelorism, proptosis, down-slanting eyes | hypertelorism, proptosis, myopia | proptosis, down-slanting eyes | hypertelorism, proptosis, down-slanting eyes, myopia | hypertelorism, proptosis | hypertelorism, proptosis |  |
| Dysmorphic features | + | + | + | + | + | + | + | + | + |  |
| Cardiac anomalies | mitral valve prolapse, aortic root dilation, vertebrobasilar and internal carotid tortuosity, dilated pulmonary artery root | - | - | mitral valve prolapse | mitral valve prolapse, mitral insufficiency, aortic root dilation | mitral valve prolapse, aortic root dilation | mitral valve prolapse, mitral insufficiency, aortic root dilation | - | - |  |
| Musculoskeletal anomalies | + | + | + | + | + | + | + | + | + |  |
| Neurological anomalies | + | + | craniosynostosis  absent | + | + | + | craniosynostosis  absent | + | + |  |
| Developmental delay | + | + | + | + | + | + | + | + | + |  |
| Other | hernias |  | hernias | hernias | hernias | hernias | hernias | hernias | hernias |  |

(d)

|  | Au, et al. [2014] | | Schepers, et al. [2016] | | | | | | | |
| --- | --- | --- | --- | --- | --- | --- | --- | --- | --- | --- |
| Patient | 30 | 31 | 32 | 33 | 34 | 35 | 36 | 37 | 38 | 39 |
| SKI gene pathogenic  variant | c.103C>T (p.Pro35Ser) | c.347G>A (p.Gly116Glu) | c.94C>G  (p.Leu32Val) | c.101G>T  (p.Glu34Val) | c.82T>A  (p.Ser28Thr) | c.103C>T  (p.Pro35Ser) | c.100G>A  (p.Gly34Ser) | c.103C>T  (p.Pro35Ser) | c.92C>T  (p.Ser31Leu) | c.289_300del  (p.Ser97_Arg100del) |
| Inheritance | de novo | de novo |  |  |  | de novo | de novo | de novo | de novo |  |
| Gender | F | M | M | F | M | F | M | F | F | M |
| Age (years) | 46 | 3 | 50 | 44 | 5 | 4 | 10 | 10 | 12 | 16 |
| Ocular findings | myopia, hypertelorism, down-slanting eyes, proptosis, right exotropia, amblyopia, retinal detachment, proliferative vitreo-retinopathy, cataracts, low vision | hypertelorism, down-slanting eyes | hypertelorism, down-slanting eyes, proptosis | hypertelorism, down-slanting eyes, proptosis | hypertelorism, down-slanting eyes, proptosis | hypertelorism, down-slanting eyes, proptosis | hypertelorism, down-slanting eyes, proptosis | hypertelorism, proptosis | hypertelorism, proptosis | hypertelorism, down-slanting eyes |
| Dysmorphic features | + | + | + | + | + | + | + | + | + | + |
| Cardiac anomalies | mitral valve prolapse | - | - |  |  | - | - | - | - | mitral valve prolapse |
| Musculoskeletal anomalies | + | + | + | + | + | + | + | + | + | + |
| Neurological anomalies | + | + | craniosynostosis absent | craniosynostosis absent | + | + |  |  | + |  |
| Developmental delay | + | + | + | + | + | + | + | + | + | + |
| Other |  | umbilical hernia |  |  |  |  |  | inguinal and umbilical hernia | feeding difficulties requiring gastrostomy, fixed talipes | bilateral hallux valgus |

(e)

|  | Schepers, et al. [2016] | | | Saito, et al. [2017] | Zhang, et al. [2019] | Choi, et al. [2019] |  |  |  |  |
| --- | --- | --- | --- | --- | --- | --- | --- | --- | --- | --- |
| Patient | 40 | 41 | 42 | 43 | 44 | 45 |  | 18 | 19 | 20 |
| SKI gene pathogenic  variant | c.101G>C  (p.Gly34Ala) | c.101G>A  (p.Gly34Asp)* | c.101G>A  (p.Gly34Asp)* | c.59C>G  (p.Thr20Arg) | c.336C>G  (p.Cys112Trp) | c.350G>A  (p.Arg117His) |  |  |  |  |
| Inheritance | de novo | GM | GM | de novo | de novo | de novo |  |  |  |  |
| Gender | M | M | F | M | F | M |  |  |  |  |
| Age (years) | 9 | 13 | 22 |  | 10 | 25 |  |  |  |  |
| Ocular findings | hypertelorism, down-slanting eyes, proptosis | hypertelorism, down-slanting eyes, shallow orbits, blepharo-phimosis | hypertelorism, down-slanting eyes, shallow orbits, blepharo-phimosis | hypertelorism, exophthalmos, exotropia |  | hypertelorism, ptosis, down-slanting eyes, myopia [-14.00, -14.75], exophoria, tilted optic nerves |  |  |  |  |
| Dysmorphic features | + | + | + | + | - | + |  |  |  |  |
| Cardiac anomalies | aortic root dilation | aortic root dilation | - | - |  | mitral valve insufficiency, dilated cardiomyopathy, aortic root dilatation, cardiomegaly, ectasia of left common iliac artery, dilatation of pulmonary trunk |  |  |  |  |
| Musculoskeletal anomalies | + | + | + | + | + | + |  |  |  |  |
| Neurological anomalies |  |  |  | + | + | + |  |  |  |  |
| Developmental delay | + | + | - | + |  |  |  |  |  |  |
| Other | neonatal intracranial hemorrhage (unclear if related to SGS) | bilateral hallux valgus | bilateral hallux valgus | motor delay, umbilical and inguinal hernia, bilateral cryptorchidism |  | bullous lung disease, gastroesophageal reflux disease |  |  |  |  |

Abbreviations: *Two siblings; GM, germline mosaicism
